# Analysis of Sensitivity, Specificity, and Predictive Values of High-Sensitivity Troponin T in a Secondary Care Setting: A Retrospective Cohort Study

**DOI:** 10.7759/cureus.44446

**Published:** 2023-08-31

**Authors:** Chinedu Orji

**Affiliations:** 1 Cardiology, North Cumbria Integrated Care, Whitehaven, GBR

**Keywords:** guidelines in medicine, myocardial infarction, cardiac troponin, diagnostic efficacy, acute chest syndrome (acs)

## Abstract

Background

High-sensitivity cardiac troponin (hs-cTn) assays have significantly improved the early detection of myocardial injury and the diagnosis of acute coronary syndrome (ACS). Different diagnostic algorithms exist for the interpretation of hs-cTn in the management of patients with suspected ACS. This study analysed the diagnostic efficacy of hs-cTn using serial and single measurements while also shedding light on the challenges associated with the use of this assay.

Methods

We reviewed 189 results belonging to 120 unique patient episodes and records for troponin tests performed in a two-week period obtained from the West Cumberland Hospital, North Cumbria Integrated Care (NCIC), Whitehaven, England. These troponin tests were carried out based on the NCIC trust guidelines for the use of troponin assays in the management of acute coronary syndrome (ACS). A positive troponin test is defined using the NCIC trust guidelines and the National Academy of Clinical Biochemistry (NACB) guidelines. The case notes of the unique patients were reviewed to determine the outcome, which is defined as the clinical diagnosis on discharge of the patient following a cardiologist review. These outcomes were then used to calculate the sensitivity, specificity, and predictive values. We also determined the alternate diagnosis for false-positive tests.

Results

Using both guidelines to assess the clinical effectiveness of the troponin assay yields slightly varying results, with the single positive test of NACB demonstrating a higher sensitivity of 92.8% (>71.4%) and a slightly better negative predictive value of 97.8% (>96%). However, using the serial troponin measurements as per the NCIC trust guideline demonstrates a better specificity of 95.2% (>42.4%) and a positive predictive value of 66% (>17.5%). False positive results are identified, which are due to alternate diagnoses such as stable angina, myocarditis, heart failure, sepsis, and malignancy.

Conclusion

High-sensitivity troponin (hs-cTn) assays play a crucial role in the early detection and management of patients with suspected ACS. This study supports evidence that serial troponin measurements are more diagnostically accurate than single troponin measurements. Although hs-cTn assays offer significant advantages, there remain challenges and limitations that require careful interpretation and clinical correlation.

## Introduction

Acute coronary syndrome (ACS) encompasses a spectrum of clinical conditions that result from reduced blood flow to the heart muscle due to compromised coronary blood supply [1]. The obstruction of coronary blood flow leads to acute myocardial ischemia and/or myocardial infarction (MI), which results from an imbalance between myocardial oxygen supply and demand. Acute coronary syndrome is characterised by three distinct clinical presentations: unstable angina, non-ST-segment elevation MI (NSTEMI), and ST-segment elevation MI (STEMI) [1]. The distinction among these subtypes is based on the presence of ischaemic symptoms, electrocardiogram (ECG) changes, and cardiac biomarkers, particularly cardiac troponins.

Conventional and high-sensitivity troponin assays

Commercial immunoassays that detect cardiac troponins in blood are now widely available to clinicians to aid in the diagnosis of ACS. Manufacturers use the terms "sensitive" and "high sensitivity" to describe the sensitivity of their assays for marketing purposes [2]. However, key factors such as sensitivity, specificity, diagnostic performance, and clinical utility determine the distinction between conventional cardiac troponin assays and high-sensitivity cardiac troponin assays.

The improved sensitivity, specificity, and diagnostic performance of high-sensitivity assays have led to their incorporation into clinical guidelines for the diagnosis and management of ACS [3,4]. They are now considered the standard of care, with conventional assays no longer recommended.

Troponin measurement is now the cornerstone of the diagnosis of MI [2]. According to the Fourth Universal Definition of MI, a collaboration between the European Society of Cardiology (ESC) and the American College of Cardiology (ACC), MI is defined by the presence of acute myocardial injury detected by abnormal cardiac biomarkers accompanied by evidence of acute myocardial ischemia [3]. According to these guidelines, the clinical criteria for the diagnosis of acute myocardial injury is the detection of a rise and/or fall of high-sensitivity troponin (hs-cTn) values, with at least one value above the 99th percentile of the upper reference limit (URL), which, if accompanied by evidence of myocardial ischemia (e.g., symptoms, ECG changes, or imaging findings), is diagnostic of acute MI. However, it must be noted that myocardial injury unrelated to ACS can be caused by non-ischaemic mechanisms such as sepsis, stroke, chronic kidney disease, heart failure, Takotsubo syndrome, and cardiomyopathy and has been associated with elevated cardiac troponin levels, especially when detected using hs-cTn assays [5].

What is a positive troponin result?

The 99th Percentile Rule

According to the National Academy of Clinical Biochemistry (NACB) guideline issued in 2007 [6], "in the presence of a clinical history suggestive of ACS, the following is considered indicative of myocardial necrosis consistent with myocardial infarction: maximal concentration of cardiac troponin value (cTn) exceeding the 99th percentile of values (with optimal precision defined by total coefficient of variation (CV) <10%) for a reference control group on at least one occasion during the first 24 hours after the clinical event." This guideline is used as one of the frameworks for determining the decision limit or a "positive" troponin result. This value varies among populations and different health institutions. For this study, the value set was levels equal to or above the test detection threshold of 12 ng/ml in North Cumbria Integrated Care (NCIC), which serves the North Cumbria population.

North Cumbria Integrated Care Trust Guideline

We will also be analysing the results based on the NCIC trust guideline, which considers troponin levels to be consistent with an MI if the first troponin is <50ng/L with a subsequent >10ng/L rise or fall on the repeat sample or the first troponin is >50ng/l with a >20% rise or fall on the repeat sample [7]. This aligns closely with the Fourth Universal Definition of MI [8], which states that "the term myocardial injury should be used when there is evidence of elevated cardiac troponin value (cTn) with at least one value above the 99th percentile upper reference limit (URL). The myocardial injury is considered acute if there is a rise and/or fall of cTn values."

However, the question arises as to what the ideal interval should be for repeating the test. There are at least six algorithms. The 0h/3h algorithm from the European guidelines and the two-hour advanced diagnostic pathway use risk score prediction tools, whereas the 0h/2h, 0h/1h European Society of Cardiology (ESC), modified 0/1h ESC, and the current US state-of-the-art algorithms (six- and 12-hour troponins) do not include risk prediction tools. No guideline supports one above the other [9]. The NCIC trust guideline advises a troponin assay on admission and a repeat test six to 12 hours later.

Aim and objectives

Most hospitals now have replaced conventional cTn tests with the new fifth-generation hs-cTn T and I assays, which can detect troponin at concentrations 10- to 100-fold lower than conventional assays [10]. Per the NCIC trust guidelines, we use the hs-cTnT assay and the Roche cTnT assay with a cutoff of 12 ng/L.

Given the variability in the diagnostic accuracy of troponin, it is of interest to continually evaluate the diagnostic and prognostic performances of this assay to ensure standards are maintained regarding the use of troponin assays in the early detection and management of ACS. In this study, we aim to calculate the sensitivity, specificity, positive predictive value (PPV), and negative predictive value (NPV) of the hs-cTnT using the two different aforementioned guidelines as a way of measuring diagnostic performance.

## Materials and methods

Study design and population

This study followed an initial departmental audit regarding the appropriate use of troponin tests in ACS management. It was a retrospective cohort study that involved analysing all troponin tests requested on admission to the emergency assessment unit and following up with the individual patients to the point of diagnosis and eventual discharge on that particular admission. The study was approved by the trust's clinical effectiveness and audit team.

We obtained a patient episode list from the NCIC trust laboratory records for troponin tests performed in the two weeks between April 1-14, 2021, on patients admitted to the emergency admission unit. We reviewed 189 results and input them into our data tool. These troponin tests belonged to 120 patient episodes at West Cumberland Hospital, Whitehaven, England.

The inclusion criteria were all patients who were admitted into the emergency admission unit and had a troponin test done within the selected two-week period. Patients who were discharged without admission from the emergency department or who did not require a troponin test were excluded from the study.

Clinical assessment

We reviewed patient information stored on the Clinical Portal, laboratory results, and post-discharge cardiologist clinic letters. We also requested patients’ medical folders from the medical records department when further information was required.

All information was collected retrospectively. We compared the initial sets of troponin tests with the eventual outcome on discharge.

Outcome measures

The primary outcome was the diagnostic performance for the rule-out of ACS at the index event for both strategies. A positive test was considered using both guidelines.

Any troponin test above the lower threshold limit in the first 24 hours; first troponin <50ng/L and >10ng/L rise or fall on repeat sample; or first troponin >50ng/L and >20% rise or fall on repeat sample

A positive outcome was a diagnosis of ACS following a cardiologist review during the current admission. We also looked into the other causes of false-positive results for these tests.

Explanation of statistical measures

When interpreting the results of a troponin assay, several statistical measures are used to evaluate its diagnostic performance [11], as briefly explained in Table 1.

**Table 1:** Explanation of statistical measures

Statistical Measure	Explanation
Sensitivity	Sensitivity refers to the ability of a troponin assay to correctly identify individuals with acute coronary syndrome (ACS). It is the proportion of true positive results (i.e., individuals with ACS correctly identified by the assay) out of the total number of individuals with ACS. A high sensitivity indicates that the troponin assay is effective at detecting ACS cases.
Specificity	Specificity refers to the ability of a troponin assay to correctly identify individuals without ACS. It is the proportion of true negative results (i.e., individuals without ACS correctly identified by the assay) out of the total number of individuals without ACS. A high specificity indicates that the troponin assay is effective at ruling out ACS in individuals who do not have the condition.
Positive Predictive Value (PPV)	The PPV is the probability that an individual has ACS when the troponin assay indicates a positive result. It takes into account both sensitivity and specificity. The PPV depends not only on the accuracy of the assay but also on the prevalence of ACS in the population being tested. If the prevalence of ACS is low, even a highly specific test may have a lower PPV.
Negative Predictive Value (NPV)	The NPV is the probability that an individual does not have ACS when the troponin assay indicates a negative result. Similar to PPV, NPV depends on both sensitivity and specificity, as well as the prevalence of ACS. A high NPV indicates that a negative result on the troponin assay can reliably rule out ACS.

It is important to note that sensitivity and specificity are inherent characteristics [12] of the test itself, whereas PPV and NPV can vary depending on the prevalence of the condition in the population being tested. Additionally, troponin assays may have different cutoff values for defining positive and negative results, which can influence the aforementioned performance measures.

Patient and public involvement

The patients or the public were not involved in the design, conduct, reporting, or dissemination plans of our research.

## Results

Diagnostic and prognostic performance

The National Academy of Clinical Biochemistry

Acute myocardial infarction (AMI) was diagnosed in 14 (3.6%) of the 120 patients reviewed using the NACB guidelines. Among the 46 patients who had negative test results, only one patient eventually had a diagnosis of ACS on discharge. Further, 13 out of the 14 patients who were diagnosed with ACS on discharge tested positive using the guideline definition. Hence, this approach had a higher sensitivity of 92.8% and an NPV of 97.8% (Table 2).

However, 61 out of the 106 patients without ACS still had a positive test, and only 13 patients among the 74 positive tests had an ACS diagnosis. This explains the lower specificity of 42.4% and PPV of just 17.5% (Table 2).

**Table 2:** The National Academy of Clinical Biochemistry guidelines ACS: acute coronary syndrome

	ACS	No ACS	Total
Positive test	13	61	74
Negative test	1	45	46
Total	14	106	120

The NCIC NHS Trust Guidelines

Using the NCIC trust guideline definition of a positive test gave a higher specificity of 95.2% and a PPV of 66%. This was because only five of the patients without ACS outcomes had a positive test, and 10 of the 15 patients with positive tests were eventually diagnosed with ACS on discharge. Interestingly, using this guideline maintained a high NPV of 96% because ACS was diagnosed in only four patients out of 105 with a negative test, but predictably had a lower sensitivity of 71.4 due to four out of 14 patients diagnosed with ACS having a negative test (Table 3).

**Table 3:** The NCIC NHS Trust guidelines ACS: acute coronary syndrome; NCIC: North Cumbria Integrated Care; NHS: National Health Service

	ACS	No ACS	Total
Positive test	10	5	15
Negative test	4	101	105
Total	14	106	120

Analysis comparing the two guidelines showed better overall diagnostic indices using the NCIC recommendation of serial measurements over the single measurement of NACB. The results as shown in Table 4 demonstrated significantly higher specificity (92.8% >71.4%) and positive predictive values (66% >17.5%) for serial troponin measurements over single measurements, with comparable negative predictive values (96% <97.8%) and lesser sensitivity (71.4% >92.8%).

**Table 4:** Comparison of NACB and NCIC guidelines NACB: National Academy of Clinical Biochemistry; NCIC: North Cumbria Integrated Care; PPV: positive predictive value; NPV: negative predictive value

	NACB	NCIC
Sensitivity (%)	92.8	71.4
Specificity (%)	42.4	95.2
PPV (%)	17.5	66
NPV (%)	97.8	96

**Figure 1:**
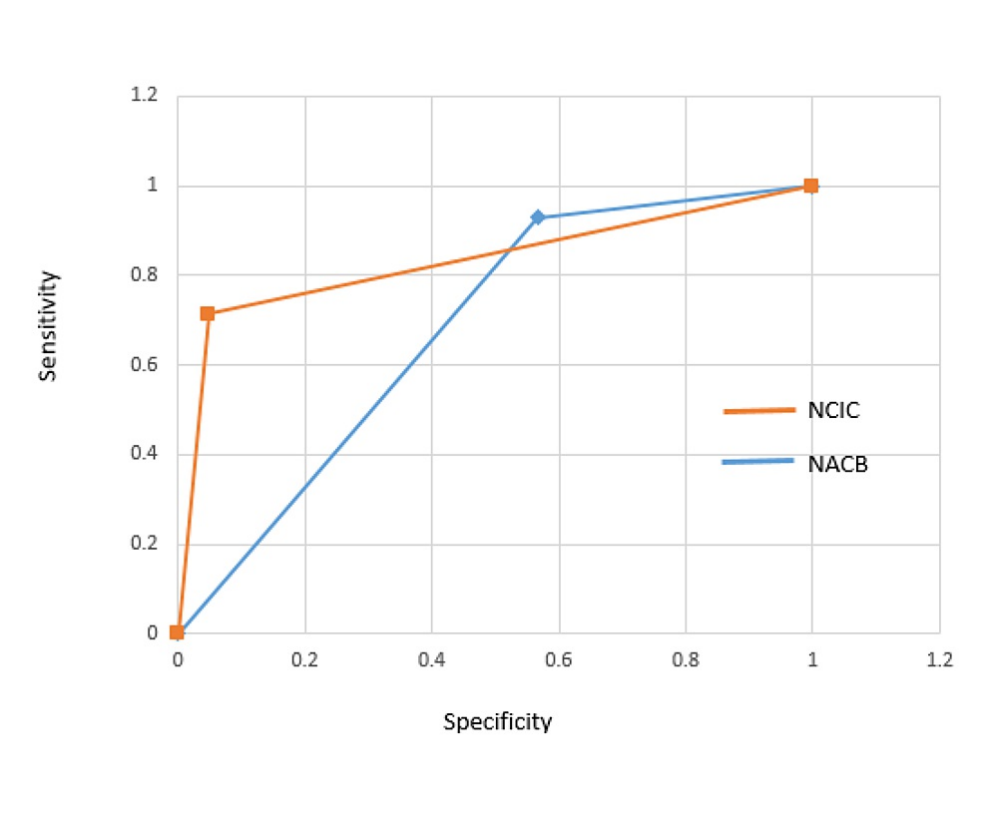
Comparison of the ROC curve and AUC of NACB and NCIC guidelines ROC curve: receiver operating characteristic curve; AUC: area under the ROC curve); NACB: National Academy of Clinical Biochemistry; NCIC: North Cumbria Integrated Care

This is well reflected in the receiver operating characteristic curve (ROC) for both parameters, as illustrated in Figure 1, with a correspondingly higher area under the ROC curve (AUC) for the NCIC guideline of 0.8330 compared to an AUC of 0.6805 for the NACB guideline.

## Discussion

High-sensitivity cardiac troponin assays have revolutionised the diagnosis and management of ACS. They have significantly improved the early detection of myocardial injury and the diagnosis of myocardial infarction (MI) compared to conventional troponin assays. These hs-cTn assays can detect very low concentrations of cardiac troponin T (cTnT) and troponin I (cTnI), allowing for more rapid diagnosis and risk stratification of patients with suspected acute coronary syndromes (ACS) [4]. Troponin measurement is now the cornerstone of the diagnosis of MI [2]. According to the Fourth Universal Definition of Myocardial Infarction, the clinical criteria for the diagnosis of MI is the presence of acute myocardial injury detected by abnormal cardiac biomarkers accompanied by evidence of acute myocardial ischaemia [3].

Several large-scale clinical trials have demonstrated the benefits of using hs-cTn assays for the diagnosis of MI and the risk stratification of patients with ACS. The High-Sensitivity Troponin in the Evaluation of Patients with Acute Coronary Syndrome (High-STEACS) trial [11] and the Biomarkers in Acute Cardiac Care (BACC) study [12] are two prominent examples that showed improved outcomes in patients managed with hs-cTn-guided strategies.

Recent developments in hs-cTn assays have focused on enhancing the analytical performance, including precision, accuracy, and reproducibility, as well as reducing the time to diagnosis. Rapid diagnostic algorithms relying on serial measurements of hs-cTn have been developed and shown to expedite the diagnosis and management of patients with suspected ACS [13]. For instance, the 0h/1h and 0h/3h algorithms relying on hs-cTn have been shown to reliably and rapidly rule in or rule out non-ST-segment elevation myocardial infarction (NSTEMI) in patients presenting with chest pain, facilitating the timely initiation of appropriate treatments [14,15].

However, new-generation high-sensitivity troponin assays (hs-cTn) still present some limitations. This has necessitated the search for novel biomarkers to rule in and rule out myocardial MI in chest pain patients early, the most notable being the heart-type fatty acid binding protein (h-FABP); however, studies on h-FABP as a reliable marker for MI diagnosis have been largely disappointing [16].

This study has raised some issues regarding troponin use in clinical management, which can impact the interpretation of results and clinical decision-making. These are listed below.

False-Positive Results

The increased sensitivity of hs-cTn assays can detect small elevations of cTn levels in conditions other than ACS. This was more noticeable when we used the NACB single measurement guideline for a positive test, which gave a high percentage of false positive results of 82.4%. This is because the troponin assay cannot differentiate between a true type I MI secondary to a plaque rupture or various other mimics of plaque, including demand ischemia [17]. In our study, we identified other conditions that led to a false positive test, such as stable angina, myocarditis, heart failure, sepsis, malignancy, pulmonary embolism, or pneumonia. This underscores the need for a comprehensive clinical evaluation that incorporates patient history, ECG findings, and other diagnostic tests.

Variability in the URL

There is variability in the 99th percentile URL among different human populations and across assays, which may impact the diagnostic accuracy. This explains the need to make sure cutoff limits are studied and designated for a specific population. This helps to accurately determine what would constitute a positive test to ensure low false positives and negatives [17,18].

Diagnostic Challenges in Early Presenters

Patients presenting very early after symptom onset may have troponin levels below the assay’s detection limit or within the reference range [17,18]. In such cases, relying solely on hs-cTn may delay the diagnosis and initiation of appropriate therapy. This observation could explain the reason why serial testing of troponin is more accurate in diagnosing ACS, as shown in the improved diagnostic indices using the trust guideline definition for a positive test, and supports the evidence for the Fourth Universal Definition of MI, which requires a rise and/or fall of high-sensitivity troponin (hs-cTn) values [3].

Interpretation of Serial Changes

The rise and/or fall of hs-cTn levels is an essential component of the diagnostic criteria for MI [3]. However, the optimal timing and threshold for significant changes in troponin levels are still under debate, which may create uncertainty in the interpretation of serial measurements [19]. This makes it vital to ensure that the patient has had chest pains for at least three hours before presentation; otherwise, a developing ACS might not be picked up, which could lead to misdiagnosis and improper management. Using risk scores such as the Global Registry of Acute Coronary Events (GRACE) and History, Electrocardiogram, Age, Risk factors, and initial Troponin (HEART) scores could also help increase sensitivity.

Analytical and Biological Variability

Despite the improved precision of hs-cTn assays, there is still some degree of analytical variability between different assays and manufacturers, which may impact the clinical interpretation of results [3]. Consequently, the mixing of measurements between different assays is discouraged.

Strengths and limitations of this study

The limitations of this study include the NCIC trust’s hs-cTnT assay cutoff of 12 ng/L. The researcher is aware that there are other troponin assays with lower cutoffs (3-5 ng/L) for which a single baseline hs-cTn T measurement improves sensitivity for AMI markedly and can be used as a rule-out test in patients presenting more than three hours after symptom onset [20]. Also, the observational cohort comprised a medium-risk population attending a secondary care emergency setting. West Cumberland Hospital is a secondary care hospital with no facility for emergency coronary angiography and with follow-on primary percutaneous coronary intervention; hence, patients assessed in the ED with STEMI were transferred directly to a tertiary centre without initial admission and thus were not part of this study. Furthermore, the study may not be adequately powered because the total number of events was low. It is also worth mentioning that the final diagnosis was based on the cardiologist’s clinical diagnosis and not necessarily objective investigation findings such as angiography.

However, despite these limitations, the study was done randomly, polling all troponin results within a set period without knowledge of the clinical information, reducing bias and increasing the internal validity of the results.

## Conclusions

The measurement of cTn is now considered the gold standard for the diagnosis of MI and plays a vital role in the risk stratification and management of patients with ACS. The study demonstrated significantly higher specificity (92.8% >71.4%) and positive predictive values (66% >17.5%) for serial troponin measurements over single measurements, with comparable negative predictive values (96% <97.8%) and lesser sensitivity (71.4% >92.8%). This supports evidence that serial troponin measurements are more diagnostically accurate than single troponin measurements. However, although hs-cTn assays offer significant advantages, there remain challenges and limitations, such as high false-positive rates, requiring careful interpretation and clinical correlation.

## References

[1] Amsterdam EA, Wenger NK, Brindis RG (2014). 2014 AHA/ACC guideline for the management of patients with non-ST-elevation acute coronary syndromes: a report of the American College of Cardiology/American Heart Association Task Force on Practice Guidelines. Circulation.

[2] Thygesen K, Mair J, Giannitsis E (2012). How to use high-sensitivity cardiac troponins in acute cardiac care. Eur Heart J.

[3] Thygesen K, Alpert JS, Jaffe AS, Chaitman BR, Bax JJ, Morrow DA, White HD (2018). Fourth universal definition of myocardial infarction (2018). Circulation.

[4] Roffi M, Patrono C (2016). CardioPulse: 'Ten Commandments' of 2015 European Society of Cardiology Guidelines for the management of acute coronary syndromes in patients presenting without persistent ST-segment elevation (NSTE-ACS). Eur Heart J.

[5] Giannitsis E, Katus HA (2013). Cardiac troponin level elevations not related to acute coronary syndromes. Nat Rev Cardiol.

[6] Mahajan VS, Jarolim P (2011). How to interpret elevated cardiac troponin levels. Circulation.

[7] Buchanan L, Shelton R (2023). Guidelines for the initial management of acute coronary syndromes (St elevation myocardial infarction, non-ST elevation myocardial infarction and unstable angina) in adults. NCIC Clinical Guidelines (v01.1.

[8] Thygesen K, Alpert JS, Jaffe AS, Chaitman BR, Bax JJ, Morrow DA, White HD (2019). Fourth universal definition of myocardial infarction (2018). Eur Heart J.

[9] Shah AS, Anand A, Strachan FE (2018). High-sensitivity troponin in the evaluation of patients with suspected acute coronary syndrome: a stepped-wedge, cluster-randomised controlled trial. Lancet.

[10] Neumann JT, Weimann J, Sörensen NA (2021). A biomarker model to distinguish types of myocardial infarction and injury. J Am Coll Cardiol.

[11] Lalkhen AG, McCluskey A (2008). Clinical tests: sensitivity and specificity. Continuing Education in Anaesthesia Critical Care & Pain.

[12] Bentley TG, Catanzaro A, Ganiats TG (2012). Implications of the impact of prevalence on test thresholds and outcomes: lessons from tuberculosis. BMC Res Notes.

[13] Keller T, Zeller T, Peetz D (2009). Sensitive troponin I assay in early diagnosis of acute myocardial infarction. N Engl J Med.

[14] Neumann JT, Sörensen NA, Rübsamen N (2019). Evaluation of a new ultra-sensitivity troponin I assay in patients with suspected myocardial infarction. Int J Cardiol.

[15] Twerenbold R, Neumann JT, Sörensen NA (2018). Prospective validation of the 0/1-H algorithm for early diagnosis of myocardial infarction. J Am Coll Cardiol.

[16] Bivona G, Agnello L, Butera D, Ciaccio M (2018). Clinical utility of HFABP in acute myocardial infarction. Acta Medica Mediterranea.

[17] Amundson BE, Apple FS (2015). Cardiac troponin assays: a review of quantitative point-of-care devices and their efficacy in the diagnosis of myocardial infarction. Clin Chem Lab Med.

[18] Garg P, Morris P, Fazlanie AL (2017). Cardiac biomarkers of acute coronary syndrome: from history to high-sensitivity cardiac troponin. Intern Emerg Med.

[19] Chapman AR, Bularga A, Mills NL (2020). High-sensitivity cardiac troponin can be an ally in the fight against COVID-19. Circulation.

[20] Narayanan MA, Garcia S (2019). Role of high-sensitivity cardiac troponin in acute coronary syndrome. US Cardiology Review.

